# Engaging Older Chinese-Speaking Cancer Patients in Support Groups: Post-pandemic Lessons Learned

**DOI:** 10.1093/geroni/igaf122.3756

**Published:** 2025-12-31

**Authors:** Qi Chen, Xiaoli Li

**Affiliations:** Hunter College - City University of New York, Hunter College - City University of New York, New York, United States; Hunter College - City University of New York, New York, New York, United States

## Abstract

Older Chinese immigrants with cancer in the U.S. face linguistic, cultural, and psychosocial barriers, posing challenges for support group engagement. The COVID-19 pandemic accelerated the adoption of online group models, creating both challenges and opportunities for care delivery. This qualitative study explored strategies for engaging Chinese-speaking older adults with cancer support groups in the post-pandemic era. Using a community-engaged approach, we recruited Chinese-speaking cancer patients, caregivers, and social workers or community workers who have experience working with older Chinese-speaking cancer patients. Semi-structured interviews were conducted in English or Mandarin, online or in person, and analyzed inductively using thematic analysis. Nineteen participants were interviewed, including patients (n = 7), one caregiver (n = 1), and practitioners (n = 11). Strategies for engaging older patients included: (1) building trust through shared language and cultural familiarity, facilitated by bilingual practitioners who understood norms around emotional restraint and family duty; (2) embedding health education in accessible, culturally grounded language to address misinformation and reduce uncertainty; (3) introducing emotional support gradually, through activities or informational sessions rather than direct questioning about distress; (4) aligning group design with family-centered values, recognizing older adults’ prioritization of family needs; and (5) leveraging online formats to reduce barriers of transportation, stigma, and disclosure. In the post-pandemic era, online group-based care presents promising avenues to reduce barriers and foster engagement among Chinese-speaking older adults with cancer. Culturally responsive strategies that build trust, deliver accessible health information, and use flexible virtual formats can enhance equitable access to psychosocial support.

